# The impact of combat sports on undergraduate students’ subjective well-being: chain mediation effects of emotional intelligence and self-esteem

**DOI:** 10.3389/fpsyg.2025.1659084

**Published:** 2025-08-26

**Authors:** Lei Ying, Qingqing Yang

**Affiliations:** ^1^School of Wushu, Chengdu Sports University, Chengdu, China; ^2^Chinese Wushu Academy, Beijing Sports University, Beijing, China

**Keywords:** combat sports, subjective well-being, emotional intelligence, self-esteem, the chain mediation effect

## Abstract

**Background:**

With the continuous development of positive psychology, undergraduate students’ subjective well-being has increasingly become a focal point for researchers. Combat sports, as an effective means of promoting mental health, have significant potential for enhancing undergraduate students’ subjective well-being, yet the underlying mechanisms warrant in-depth investigation. This study aimed to explore the impact of combat sports on undergraduate students’ subjective well-being and examine the chain mediation effects of emotional intelligence and self-esteem.

**Methods:**

This study employed a cross-sectional design to collect data from undergraduate students participating in combat sports across 10 universities in Sichuan Province, China, through questionnaire surveys. The research instruments included the Physical Activity Rating Scale, Subjective Well-being Scale, Emotional Intelligence Scale, and Self-esteem Scale to comprehensively assess participants’ psychological and behavioral characteristics. Following data collection, statistical analysis was conducted using SPSS 26.0, with structural equation modeling (AMOS) and Bootstrap methods employed to examine potential mediation effects and ensure reliability of the findings.

**Results:**

The analysis revealed significant positive correlations among combat sports participation, subjective well-being, emotional intelligence, and self-esteem. Specifically, combat sports demonstrated a significant direct effect on undergraduate students’ subjective well-being, indicating that participation in combat sports directly enhances individuals’ well-being levels. Furthermore, emotional intelligence and self-esteem exhibited chain mediation effects between combat sports and subjective well-being, whereby combat sports indirectly influenced subjective well-being by enhancing individuals’ emotional intelligence and self-esteem.

**Conclusion:**

Combat sports not only directly predict undergraduate students’ subjective well-being but also indirectly influence subjective well-being through the psychological mediating variables of emotional intelligence and self-esteem. This study elucidates the underlying mechanisms linking combat sports participation and undergraduate students’ subjective well-being, providing empirical support and theoretical foundation for enhancing undergraduate students’ subjective well-being and maintaining their mental health through combat sports participation.

## 1 Introduction

With the rapid expansion of higher education in China, mental health concerns among undergraduate students have become increasingly salient. According to the “2022 National Survey Report on College Students’ Mental Health Status,” the prevalence rates of depression and anxiety among Chinese undergraduate students reached 21.48 and 45.28%, respectively (Institute of Psychology of CAS, 2023). Mental health disorders among undergraduate students have emerged as a critical challenge within educational and public health domains. Contemporary undergraduate students commonly encounter multifaceted stressors including academic pressure, employment competition, and interpersonal adjustment difficulties. These psychosocial stressors frequently contribute to the manifestation of psychological disorders, particularly anxiety and depressive symptomatology. Subjective well-being, defined as an individual’s comprehensive subjective evaluation of life quality based on personal standards, encompasses two primary dimensions: a cognitive component (life satisfaction) and an affective component (the balance between positive and negative affect) (Diener et al., 2009). Compared to singular mental health indicators, subjective well-being demonstrates distinct advantages. First, it provides a more comprehensive framework for psychological functioning assessment, capable of simultaneously capturing negative indicators emphasized by traditional pathological models and positive indicators highlighted by positive psychology (Suldo and Shaffer, 2008). Second, as a comprehensive indicator reflecting overall psychological functioning and life adaptation, subjective well-being enables more accurate evaluation of whether undergraduate students successfully navigate core developmental challenges (Renshaw and Bolognino, 2016). Finally, extensive research demonstrates that subjective well-being exhibits significant predictive validity for individuals’ future academic achievement, career development, and interpersonal relationship quality (Bücker et al., 2018; Cárdenas et al., 2022; Zhang et al., 2024), with its enhancement often accompanied by concurrent improvement across multiple mental health indicators (Carr et al., 2021). Consequently, enhancing subjective well-being not only ameliorates undergraduate students’ mental health status but also holds substantial implications for their academic performance, social adaptation, and future development. However, traditional mental health interventions have demonstrated limited efficacy within undergraduate populations, necessitating the exploration of more targeted and effective enhancement pathways.

In response to multifaceted challenges including academic pressure, employment competition, and interpersonal adjustment difficulties, the Chinese government promulgated the “Special Action Plan for Comprehensively Strengthening and Improving Student Mental Health Work in the New Era (2023-2025),” which explicitly advocates for “actively utilizing physical exercise and other modalities to promote student mental health” (Ministry of Education of China, 2023). Physical exercise, as an effective means of promoting both physical and mental health, has demonstrated well-established positive effects on psychological well-being (Martín-Rodríguez et al., 2024; Eather et al., 2023). Within the Chinese cultural context, combat sports constitute a distinctive category of confrontational athletic activities that extend beyond physical movement and strength training to emphasize the cultivation of psychological concentration and emotional regulation capabilities (Vertonghen and Theeboom, 2010). The philosophical principles of self-cultivation and harmonious integration of internal and external development inherent in combat sports demonstrate remarkable congruence with traditional mind–body harmony concepts, conferring unique cultural adaptability advantages in mental health promotion (Barreira, 2019). Compared to conventional sports modalities, combat sports exhibit significant superiority in fostering individual self-confidence, stress tolerance, and emotional regulation skills (Ciaccioni et al., 2025; Kim and Cruz, 2021). Participants encounter highly uncertain confrontational scenarios requiring instantaneous decision-making under physical contact pressure while directly experiencing the psychological impact of competitive outcomes (Quan et al., 2025). This distinctive athletic environment necessitates not only robust physical competencies but also the development of corresponding psychological adaptation capacities to navigate complex emotional and cognitive challenges (Mojtahedi et al., 2023). Based on these distinctive characteristics, a meta-analytic investigation conducted by Moore et al. (2020) demonstrated that combat sports training exerts significant positive effects on subjective well-being. Research demonstrates that appropriate participation in combat sports can alleviate mental tension and stress, foster positive coping strategies, and promote the generation of optimistic emotions (Moore et al., 2019), thereby reducing anxiety and depressive symptoms while directly enhancing subjective well-being.

Among the numerous psychological variables that may influence subjective well-being, emotional intelligence and self-esteem are recognized as two fundamental constructs. Emotional intelligence refers to the comprehensive ability to accurately perceive, understand, express, and regulate emotions in oneself and others, while utilizing emotional information to guide thinking and behavior. It encompasses four core dimensions: emotion perception, emotion understanding, emotion integration, and emotion regulation (Salovey and Mayer, 1990). According to Gross’s emotion regulation theory, emotional intelligence represents not only a crucial capability for individual adaptation to complex social environments but also a pivotal factor in maintaining psychological equilibrium within high-stress, high-challenge contexts (Gross, 2015). Within the domain of sports, particularly in confrontational athletics, participants are required to maintain emotional stability in high-pressure competitive environments while precisely identifying and regulating their emotional states during dynamic interactions, thereby continuously enhancing their emotional perception, comprehension, and regulation capabilities. This provides an authentic stress-testing environment for emotional intelligence development (Castro-Sánchez et al., 2018). Existing research demonstrates that combat sport athletes exhibit higher emotional intelligence compared to non-athletes (Costarelli and Stamou, 2009), with combat sports significantly enhancing individuals’ emotional intelligence (Bae and Roh, 2021). Individuals with high emotional intelligence demonstrate greater effectiveness in identifying and managing negative emotions, maintaining positive and stable emotional states, and significantly improving subjective well-being (Extremera et al., 2020).

Self-esteem, as an individual’s overall evaluation of self-worth, serves as an important protective factor for mental health (Yu et al., 2012). The developmental trajectory of self-esteem theory demonstrates that individual self-worth is not a static cognitive structure but rather a psychological trait dynamically constructed through environmental interactions (Rosenberg, 1965). Combat sports provide optimal contextual conditions for self-esteem development, enabling participants to accumulate positive achievement experiences and favorable feedback through skill enhancement, challenge management, team collaboration, and social recognition, thereby elevating self-identity and self-worth evaluation while strengthening self-esteem levels (Healey et al., 2025). Research indicates that combat sports significantly enhance individual self-esteem levels. Undergraduate students with high self-esteem possess stronger self-identity and psychological adaptation capabilities, demonstrate greater tendency to adopt positive coping strategies when facing challenges, and consequently experience higher life satisfaction and subjective well-being (Gonzales-Backen et al., 2015). Notably, a sequential relationship may exist between emotional intelligence and self-esteem, whereby emotional intelligence, as a fundamental emotional management capacity, enables individuals to better handle emotional conflicts and maintain interpersonal relationships, thereby gaining more successful experiences and social recognition, which subsequently enhances self-esteem levels (Cheung et al., 2015; Rey et al., 2011). However, empirical validation of this hypothesis within combat sports contexts remains lacking. The mechanisms by which emotional intelligence and self-esteem function in the process of combat sports influencing subjective well-being, along with their underlying psychological mechanisms and pathway relationships, have not yet received systematic empirical examination.

Existing research has predominantly focused on physiological changes following combat sports participation (Linhares et al., 2023; Kotarska et al., 2019; Durkalec-Michalski et al., 2016), while studies examining the psychological mechanisms of combat sports have largely concentrated on direct effect verification (Andreato et al., 2022), lacking in-depth exploration of psychological mechanisms. Specifically, the underlying mechanisms of key mediating variables such as emotional intelligence and self-esteem remain systematically unverified. Based on this research gap, the present study employs combat sports as an entry point to thoroughly investigate their impact mechanisms on undergraduate students’ subjective well-being, with particular emphasis on analyzing the chain mediation effects of emotional intelligence and self-esteem. This research not only contributes to filling theoretical gaps in combat sports psychological benefits research and elucidating the intrinsic mechanisms through which combat sports promote mental health, but also provides scientific theoretical foundations and practical guidance for university mental health education and physical education reform. The study holds significant theoretical value and practical implications for enhancing undergraduate students’ subjective well-being and promoting their comprehensive development.

## 2 Research statement

This study is grounded in Self-Determination Theory (SDT) as its theoretical foundation. The theory posits that individuals possess three basic psychological needs: autonomy, competence, and relatedness. When these needs are adequately satisfied, individuals’ intrinsic motivation is activated, thereby promoting mental health and enhancing subjective well-being (Teixeira et al., 2012). Self-Determination Theory provides an important theoretical framework for understanding the relationships among combat sports, emotional intelligence, self-esteem, and subjective well-being. Specifically, combat sports can effectively satisfy undergraduate students’ three basic psychological needs: autonomy needs are fulfilled through participants’ autonomous selection of training content and tactical strategy formulation (Mossman et al., 2024); competence needs are realized through skill enhancement, achievement of training objectives, and improvement in competitive performance (Tarver and Levy, 2023); relatedness needs are satisfied through interactions with coaches and teammates and the establishment of team belonging (Cao and Lyu, 2024).

**Figure 1:**
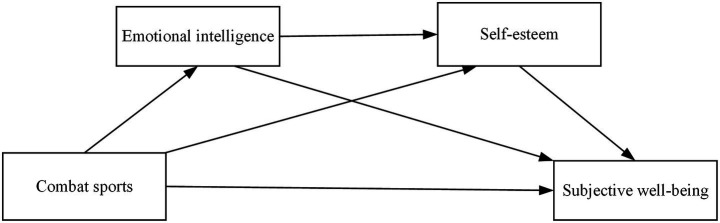
A hypothetical model of combat sports affecting subjective well-being.

The satisfaction of basic psychological needs further promotes the development of emotional intelligence. In combat training and competition, participants must make autonomous decisions under high-pressure environments, requiring acute emotional perception and regulation abilities. Simultaneously, interactions with teammates and opponents facilitate the development of abilities to understand and manage others’ emotions, thereby comprehensively enhancing emotional intelligence levels. The enhancement of emotional intelligence forms a positive cycle with self-esteem development (Carr, 2000). When individuals can effectively utilize emotional intelligence to address challenges in combat situations, they not only strengthen positive cognitions about their own abilities but also more readily gain recognition and support from coaches and teammates, significantly enhancing self-esteem levels. Ultimately, individuals with high self-esteem maintain firm beliefs in their self-worth and demonstrate greater tendency to approach life challenges with positive attitudes, thereby effectively enhancing subjective well-being (Katsantonis et al., 2023). Based on the above theoretical analysis, this study constructs a chain mediation model of combat sports influencing undergraduate students’ subjective well-being through emotional intelligence and self-esteem (as shown in Figure 1) and proposes corresponding research hypotheses accordingly.

## 3 Hypotheses development

### 3.1 The relationship between combat sports

Combat sports, as comprehensive forms of exercise integrating physical training, skill acquisition, and psychological conditioning, have demonstrated positive effects on individuals’ subjective well-being (Ciaccioni et al., 2024). This study focuses on three combat sports with high prevalence in Chinese universities: Wushu Sanda, Taekwondo, and Kickboxing. These disciplines possess strong appeal and participation rates among undergraduate students due to their distinctive cultural backgrounds and technical characteristics. According to the core postulates of SDT, when individuals’ three basic psychological needs—autonomy, competence, and relatedness—are satisfied, this directly promotes mental health and subjective well-being enhancement (Deci and Ryan, 2000). Specifically, combat sports require participants to autonomously select tactical strategies, flexibly adjust technical movements, and actively control competitive rhythm during confrontational processes. This high degree of autonomous decision-making directly satisfies individuals’ intrinsic need for autonomy.

Existing research demonstrates that during high-intensity combat sports participation, the body secretes substantial quantities of neurotransmitters including endorphins, dopamine, and serotonin (Tanriverdi et al., 2013), which effectively alleviate pain, reduce stress, and induce feelings of pleasure and relaxation, thereby enhancing individual motivation and satisfaction while reducing the probability of negative emotions such as depression and anxiety (Lee et al., 2021). Concurrently, combat sports feature distinct skill progression systems and immediate feedback mechanisms. Through the integration of high-intensity aerobic and anaerobic training, participants experience significant improvements in cardiopulmonary endurance, muscular strength, explosive power, and physical coordination (Santos-Junior and Franchini, 2021; Origua Rios et al., 2018). These enhancements in physical functioning not only contribute to physical fitness improvement (Fong and Ng, 2011) but also elevate individuals’ positive cognition regarding their bodily capabilities, with the mastery process from fundamental movements to complex tactics providing participants with abundant competence experiences. Furthermore, although combat sports manifest as individual disciplines, their training processes are highly dependent upon instructor-student interactions, peer support, and team cultural identification. Particularly within the Chinese cultural context, combat sports disciplines such as Wushu sanda carry profound cultural connotations, with participants acquiring strong cultural belongingness during the learning process. Consequently, this study selects Wushu Sanda, Taekwondo, and Kickboxing as combat sports for undergraduate participation, all of which can positively influence subjective well-being. Based on the preceding analysis, we propose the following hypothesis:

*Hypothesis 1*: Combat sports demonstrate a positive correlation with undergraduate students' subjective well-being.

### 3.2 The mediating role of emotional intelligence

Between combat sports participation and undergraduate students’ subjective well-being, emotional intelligence serves as a crucial mediating factor. SDT theory emphasizes that the satisfaction of basic psychological needs constitutes a dynamic process requiring individuals to possess corresponding psychological resources and regulatory capacities to maintain and facilitate this process (Curren and Park, 2024). Emotional intelligence, as individuals’ comprehensive ability to identify, understand, regulate, and utilize emotional information, represents an essential psychological resource for maintaining basic psychological need satisfaction (Mayer and Salovey, 1993). Combat sports, as highly structured confrontational physical activities, create authentic learning contexts that foster emotional intelligence development. First, high-intensity confrontation requires athletes to rapidly identify emotional cues from both themselves and opponents under pressure conditions, thereby strengthening emotional perception abilities (Janelle et al., 2020). Second, the close association between technical movements and emotional states enables athletes to understand the causal relationships among emotions, behaviors, and outcomes, deepening emotional understanding (Jekauc et al., 2021). Finally, the immediate feedback mechanisms inherent in competition allow athletes to continuously adjust their emotional regulation strategies, enhancing emotional management capabilities through the process of withstanding pressure. Empirical research demonstrates that systematic combat training significantly enhances participants’ emotional intelligence levels (Fernández et al., 2020).

The enhancement of emotional intelligence provides crucial safeguards for the sustained satisfaction of basic psychological needs and promotes subjective well-being improvement through optimizing the need satisfaction process. Specifically, in the cognitive dimension, emotional intelligence facilitates the formation of objective and positive self-evaluations, reduces emotion-induced cognitive biases, and subsequently improves life satisfaction (Palmer et al., 2002). In the affective dimension, high levels of emotional intelligence enhance individuals’ capacity to generate and maintain positive emotions while improving the regulation efficiency of negative emotions, thereby optimizing the quality and balance of emotional experiences (Szczygieł and Mikolajczak, 2017). In the social dimension, emotional intelligence promotes high-quality interpersonal interactions, expands social support networks, and provides external resource security for well-being (Olasupo et al., 2021). Therefore, emotional intelligence plays a pivotal mediating role in the process by which combat sports promote subjective well-being among undergraduate students. Based on this theoretical analysis, this study proposes:

*Hypothesis 2*: Emotional intelligence mediates the relationship between combat sports and undergraduate students' subjective well-being.

### 3.3 The mediating role of self-esteem

Between combat sports participation and undergraduate students’ subjective well-being, self-esteem serves as an important mediating factor. Self-esteem refers to individuals’ cognitive and emotional responses to their own physical attributes, personality characteristics, social status, and behaviors (Sonstroem and Morgan, 1989), serving as a crucial psychological factor in predicting emotional states and life transitions. Within the SDT theoretical framework, self-esteem originates from the satisfaction of basic psychological needs, reflecting individuals’ confidence in their intrinsic worth rather than dependence on external evaluations or social comparisons (Ryan and Deci, 2000). When combat sports satisfy undergraduate students’ basic psychological needs, they directly promote self-esteem development. On one hand, muscular strength demonstrates positive associations with individual physical self-esteem, emotional stability, extraversion, and self-confidence (Slimani and Chéour, 2016), with strength training significantly enhancing individual self-cognition and positivity. On the other hand, combat sports can alleviate depressive symptoms through elevating physical self-esteem levels, exerting significant promotional effects on undergraduate students’ positive emotions, self-confidence, and life satisfaction (Nie et al., 2025). Ultimately, combat sports provide individuals with abundant success experiences through skill mastery, challenge management, and goal achievement, effectively enhancing self-efficacy and self-worth cognition. These positive experiences based on intrinsic need satisfaction collectively construct self-esteem that is independent of external comparisons.

According to SDT theory, self-esteem, as a direct consequence of basic psychological need satisfaction, can stably predict subjective well-being, multiple studies demonstrate that self-esteem exerts a positive predictive effect on subjective well-being (Duy and Yıldız, 2019). High self-esteem indicates that individuals maintain positive attitudes toward their own abilities and worth, not only enhancing their capacity to cope with adversity but also serving as a robust predictor of subjective well-being. Individuals with higher and more stable self-esteem experience elevated levels of subjective well-being (Zell and Johansson, 2025). As a vital psychological resource for undergraduate students, self-esteem effectively predicts their subjective well-being and functions as a protective factor promoting positive emotional development. Previous research has found that high self-esteem significantly and positively predicts undergraduate students’ subjective well-being, with characteristics such as self-worth, autonomy concepts, and sense of control proving crucial for enhancing subjective well-being. Individuals who tend to possess greater dignity, internal locus of control preferences, and fewer internal conflicts are more likely to experience happiness (Çiçek, 2021). Therefore, self-esteem plays a significant mediating role in the process by which combat sports promote subjective well-being among undergraduate students. Based on this theoretical foundation, this study proposes:

*Hypothesis 3*: Self-esteem mediates the relationship between combat sports and undergraduate students' subjective well-being.

### 3.4 The chain mediation role of emotional intelligence and self-esteem

Emotional intelligence and self-esteem, as two pivotal psychological variables, may exert a serial mediating effect in the process through which combat sports influence subjective well-being among university students. Within the framework of SDT, emotional intelligence and self-esteem assume distinct functional roles in the satisfaction of basic psychological needs (Ryan and Deci, 2024), exhibiting specific developmental sequences that establish a mutually reinforcing serial developmental relationship (Roth et al., 2019). From a theoretical mechanism perspective, combat sports promote emotional intelligence development through the satisfaction of basic psychological needs. As previously discussed, the emotional regulation demands inherent in high-intensity confrontational contexts provide systematic training opportunities for emotional intelligence enhancement. When emotional intelligence is effectively developed, individuals acquire crucial cognitive-emotional resources for maintaining and optimizing need satisfaction processes, enabling more stable fulfillment of autonomy, competence, and relatedness needs across various contexts (Van den Broeck et al., 2016). This stabilization of need satisfaction further creates favorable conditions for self-esteem development. Specifically, the facilitative effect of emotional intelligence on self-esteem development manifests across multiple dimensions. First, through enhanced emotion regulation capabilities, individuals acquire an effective sense of control over their psychological states, thereby strengthening autonomy experiences. Second, through the mastery of emotional management skills, individuals can maintain effective functional states across diverse contexts, significantly enhancing competence experiences. Third, through the development of emotional communication abilities, individuals gain access to higher-quality social interactions and support, effectively strengthening relatedness experiences. The accumulation and stabilization of these need satisfaction experiences facilitated by emotional intelligence ultimately promote self-esteem development based on intrinsic value confirmation.

Self-esteem, as a stable psychological resource, further facilitates the enhancement of subjective well-being. Individuals with high self-esteem tend to engage in more positive interpretations of life events, demonstrate greater attention to positive aspects of life, and maintain optimistic attitudes toward the future (Baumeister et al., 2003). This positive cognitive tendency directly influences life satisfaction, which constitutes the cognitive component of subjective well-being. Simultaneously, elevated self-esteem levels also affect individuals’ emotional experience patterns, facilitating greater experiences of positive emotions (such as happiness, contentment, and pride) while reducing experiences of negative emotions (such as anxiety, depression, and shame) (Han et al., 2025). This positive emotional bias contributes to significant improvements in college students’ subjective well-being. This chain process comprehensively exemplifies the developmental mechanism of “environmental support → need satisfaction → psychological resource development → subjective well-being enhancement” within SDT. Throughout this process, emotional intelligence functions as a procedural resource that facilitates the maintenance of need satisfaction, while self-esteem operates as an outcome resource that directly promotes subjective well-being experiences. These two components work synergistically to collectively construct the complete pathway through which combat sports promote psychological health development in college students. Based on this theoretical analysis, this study proposes:

*Hypothesis 4*: Emotional intelligence and self-esteem demonstrate chain mediation effects between combat sports and undergraduate students' subjective well-being.

## 4 Materials and methods

### 4.1 Research design

To obtain the primary data required for this study, researchers designed a self-administered questionnaire based on existing literature and validated data collection instruments, focusing on evaluating undergraduate students’ combat sports participation, emotional intelligence, self-esteem, and subjective well-being. Prior to conducting the final survey, the research team conducted a preliminary assessment with 30 participants (pilot test) to ensure questionnaire feasibility and test response rates. Through the pilot study, researchers made adjustments and modifications to specific questionnaire items to maximize response accuracy and comprehensiveness. From an economic development perspective, Chengdu ranks among the forefront of Chinese cities and serves as a model in urbanization processes, having consecutively topped China’s Happiest Cities rankings for 13 years. The research team conducted questionnaire surveys among undergraduate students participating in combat sports across 10 universities in Sichuan Province, China, from December 25, 2024, to May 25, 2025. The questionnaire content included: Physical Activity Rating Scale, Subjective Well-being Scale, Emotional Intelligence Scale, and Self-esteem Scale. All data collection was conducted under standardized conditions to minimize data bias. Each questionnaire required approximately 10–20 min to complete, and all invited participants participated voluntarily. The Ethics Committee of Chengdu Sport University approved this project ([2024]164), and the research process complied with ethical requirements outlined in the Declaration of Helsinki.

### 4.2 Inclusion and exclusion criteria

The inclusion criteria for study participants were: (1) current full-time undergraduate students; (2) documented combat sports training experience with continuous training duration ≥6 months and current maintenance of regular training at least twice weekly; (3) adequate Chinese reading comprehension abilities to independently complete questionnaire surveys; (4) good physical and mental health status with no history of severe mental illness or current mental health issues; (5) voluntary participation with full understanding of research objectives, procedures, and risks, with signed informed consent.

The exclusion criteria for participants were: (1) involuntary participation or requests to withdraw from the study during the survey process; (2) presence of severe cognitive impairments, mental illness, or current psychological treatment that might affect the authenticity and validity of questionnaire responses; (3) history of serious sports injuries or medical restrictions within the past 6 months affecting normal training participation; (4) poor questionnaire response quality, including: incorrect or missing responses to key items, obvious patterned response behaviors, or abnormal completion times.

### 4.3 Measurement tool design and reliability testing

#### 4.3.1 Combat sports activity level

The Physical Activity Rating Scale-3 (PARS-3), developed by Liang (1994), was utilized to assess participants’ combat sports activity levels. The PARS-3 comprises three dimensions: exercise intensity, exercise duration, and exercise frequency. Each item employs a 5-point Likert scale with corresponding scores ranging from 1 to 5. The total scale score is calculated using the formula: frequency score × (duration score –1) × intensity score, with a possible range of 0–100. Higher scores indicate greater exercise intensity, frequency, and duration. Physical activity level classification criteria are: ≤19 points for low physical activity; 20–42 points for moderate physical activity; ≥43 points for high physical activity. In this study, the scale demonstrated a Cronbach’s *α* coefficient of 0.794.

#### 4.3.2 Subjective well-being scale

This study employed the Subjective Well-being Scale developed by Diener (1984). The scale comprises two subscales: the Satisfaction with Life Scale and the Positive and Negative Affect Schedule. The Satisfaction with Life Scale contains 5 items utilizing a 7-point scoring system (1 = strongly disagree, 7 = strongly agree). The Positive and Negative Affect Schedule includes 14 items total, with the positive affect dimension encompassing 8 items (items 1, 4, 6, 8, 9, 11, 12, 14) and the negative affect dimension containing 6 items (items 2, 3, 5, 7, 10, 13). These items employ a 4-point scoring system (1 = not at all, 4 = very often). The final subjective well-being score is calculated as the sum of the life satisfaction score and the positive and negative affect scores (with negative affect items reverse-scored). Higher scores indicate greater subjective well-being. In this study, the scale demonstrated a Cronbach’s *α* coefficient of 0.934.

#### 4.3.3 Emotional intelligence scale

The Emotional Intelligence Scale is a self-report emotional intelligence scale developed by Schutte et al. (1998). based on Mayer and Salovey’s emotional intelligence theory The scale contains 33 items across four dimensions: emotion perception, self-emotion regulation, others-emotion regulation, and emotion utilization. Items 2, 3, 9, 12, 13, 14, 16, 22, 23, 28, 30, and 31 assess emotion perception; items 7, 8, 10, 17, 20, and 24 assess self-emotion regulation; items 4, 6, 11, 18, 19, 21, 26, 27, 29, and 32 assess others-emotion regulation; and items 1, 5, 15, 25, and 33 assess emotion utilization. Emotional intelligence assessment employs a 5-point Likert scale (1 = completely disagree, 5 = completely agree), with a total possible score of 165 points. Items 5, 28, and 33 are reverse-scored. Higher emotional intelligence scores indicate greater emotional intelligence levels in respondents, while lower scores indicate lower emotional intelligence levels. In this study, the scale demonstrated a Cronbach’s α coefficient of 0.959.

#### 4.3.4 Self-esteem scale

Self-esteem was assessed using the Rosenberg Self-Esteem Scale (RSES) developed by Rosenberg (1965). The scale comprises 10 items, with five items requiring reverse scoring (items 3, 5, 8, 9, and 10). Responses were recorded on a 4-point Likert scale ranging from 1 (“strongly disagree”) to 4 (“strongly agree”). Higher total scores indicate higher levels of self-esteem. The Cronbach’s α coefficient for this scale in the present study was 0.910.

#### 4.3.5 Control variables

To validate the effects of combat sports on undergraduate students’ subjective well-being, this study selected the following control variables based on relevant theories and empirical research to maximize internal validity and minimize interference from potential confounding factors. Gender was included as an important demographic control variable (male = 1, female = 0). Gender differences hold significant implications in subjective well-being research, with effects primarily manifested through differences in gender role socialization, emotional expression patterns, and coping strategies (Batz-Barbarich et al., 2018). Research by Matud et al. (2019) demonstrated that males scored higher on self-acceptance and autonomy dimensions, while females exhibited superior performance in personal growth and interpersonal relationships dimensions. Although effect sizes were relatively small (Cohen’s d = 0.1–0.3), they achieved statistical significance in large-sample studies, necessitating control. Only-child status possesses particular importance within the Chinese cultural context, employing binary coding (only child = 1, non-only child = 0). Based on Resource Dilution Theory, only children receive complete family resource investment but simultaneously bear greater achievement expectation pressures and future caregiving responsibilities. The psychological stress risk for non-only children significantly exceeds that of only children (Lu, 2016), making this variable crucial for accurately estimating combat sports effects. Geographic origin reflects the influence of urban–rural background differences on undergraduate adaptation and well-being, employing binary coding (urban = 1, rural = 0). Students from rural backgrounds face multiple challenges including economic capital, social-cultural capital, and environmental adaptation when transitioning to urban university environments (Yu et al., 2022). Geographic origin differences may confound the relationship between exercise participation and well-being by influencing educational resource access, cultural adaptation, and social support networks. Academic year reflects the developmental characteristics of undergraduate students at different stages, processed using dummy coding with freshmen as the reference group to establish three dummy variables (sophomore = 1, others = 0; junior = 1, others = 0; senior = 1, others = 0). Based on student development theory and psychosocial development models, undergraduate students across different academic years face significantly different developmental tasks and stressors. A 4-year longitudinal study by Shek et al. (2017) of 434 Hong Kong students revealed that undergraduate well-being demonstrates a U-shaped developmental trajectory, with sophomores experiencing a nadir characterized by highest academic pressure and lowest psychological well-being. Therefore, controlling for academic year variables holds significant importance for distinguishing natural developmental changes from combat sports intervention effects. The selection of these control variables follows best practices in subjective well-being research, ensuring accurate identification of the unique contributions of combat sports to subjective well-being through emotional intelligence and self-esteem mediation pathways. All control variables will be simultaneously incorporated into regression models and structural equation models in subsequent analyses to enhance the internal validity of research conclusions and the reliability of causal inferences.

#### 4.3.6 Statistical analysis

Following data collection and validation, all valid questionnaire data were analyzed using SPSS 26.0 software. Correlation analysis and linear regression analysis were employed to examine the effects of combat sports, emotional intelligence, and self-esteem on undergraduate students’ subjective well-being. The Amos 24.0 software package was utilized for model validation and assessment of the structural validity of the scales. Currently, the Bootstrap method represents the most commonly employed approach for testing mediation effects. This method conducts repeated sampling based on the original sample and examines the significance of mediation effect coefficients through 95% confidence intervals. Therefore, this study employed the Bootstrap method to test whether emotional intelligence and self-esteem demonstrate mediation effects between combat sports and undergraduate students’ subjective well-being, as well as whether emotional intelligence and self-esteem exhibit chain mediation effects between combat sports and undergraduate students’ subjective well-being.

## 5 Results

### 5.1 Validity testing

Since this study employed scales adapted from previous research questionnaires, validation of the scales’ reliability and validity was required. To further examine the convergent validity and reliability of the scales, Average Variance Extracted (AVE) and Construct Reliability (CR) were employed as assessment parameters. AVE is commonly used to reflect the convergent validity of scales, directly indicating the proportion of variance explained by latent variables that originates from measurement error. Higher AVE values indicate a greater percentage of variance in measurement variables explained by latent variables, correspondingly reducing measurement error. CR reflects whether all items within each latent variable consistently explain that latent variable. As shown in Table 1, the AVE values for all factors exceeded 0.5, demonstrating good model convergence. The CR values for all factors exceeded 0.7, confirming that items within each scale consistently explain their respective latent variables, indicating satisfactory construct reliability for the aforementioned scales. In summary, the questionnaire survey in this study demonstrates high reliability and validity.

**Table 1:** Validity and reliability test of the questionnaires.

Variable	CR	AVE
CS	0.794	0.562
SWB	0.834	0.627
EI	0.841	0.574
SE	0.910	0.502

CR, composite reliability; AVE, average variance extracted; CS, combat sports; SWB, subjective well-being; EI, emotional intelligence; SE, self-esteem.

### 5.2 Common method variance testing

To mitigate common method variance, coded anonymous assessment procedures were implemented during data collection to procedurally control sources of common method bias. Simultaneously, Harman’s single-factor test was conducted using SPSS 26.0 to perform exploratory factor analysis on all measurement items. Results revealed nine factors with eigenvalues greater than 1, with the first factor explaining 31.644% of the variance, which falls below the critical threshold of 40%. These findings indicate that serious common method variance issues are not present in this study.

### 5.3 Descriptive statistics and correlation analysis

This study conducted a questionnaire survey of 1,351 undergraduate students across 10 universities in Sichuan Province from December 2024 to May 2025, yielding 1,304 valid responses. As shown in Table 2, participants demonstrated balanced gender distribution, with 622 males (47.6%) and 682 females (52.3%). Regarding family composition, 448 participants (34.3%) were only children, while 856 participants (65.6%) had siblings. Geographic distribution revealed 644 participants (49.3%) from urban areas and 660 participants (50.6%) from rural areas. Academic year distribution included 532 freshmen (40.7%), 466 sophomores (35.7%), 156 juniors (11.9%), and 150 seniors (11.5%).

**Table 2:** Participant demographics.

Demographic category	Frequency	Percent%
Gender		
Male	622	47.6
Female	682	52.3
Are you an only child?		
Yes	448	34.3
No	856	65.6
Place of origin	239	22
Urban area	45	4.1
Rural area	52	4.8
Education system		
Freshman year	89	8.18
Sophomore year	185	17.01
Junior year	203	18.67
Senior year	259	23.82

This study focused on examining the overall scores of each model indicator, without further investigating the sub-dimensions of each indicator, utilizing the mean scores of each variable for analysis. The descriptive statistical results for the four variables of combat sports, emotional intelligence, self-esteem, and undergraduate students’ subjective well-being are presented in Table 3. To assess the normality of data distribution, the table includes means, standard deviations, skewness, and kurtosis values. According to the criteria proposed by Kline (2023), data can be considered to satisfy the normality assumption when the absolute values of skewness are less than 3 and the absolute values of kurtosis are less than 10. As demonstrated in Table 3, considering both skewness and kurtosis indicators, all major variables in this study satisfy the normality assumption, supporting the utilization of subsequent parametric statistical analyses, including Pearson correlation analysis and structural equation modeling. This study focused on examining the overall scores of each model indicator rather than investigating the sub-dimensions of individual indicators, employing mean scores of each variable for Pearson correlation analysis. Table 3 presents the correlation analysis results for the four variables: combat sports, emotional intelligence, self-esteem, and undergraduate students’ subjective well-being. The results revealed significant positive correlations among all pairs of the four variables. Specifically, correlation analysis demonstrated that combat sports was significantly correlated with students’ subjective well-being (*r* = 0.534, *p* < 0.01); combat sports showed significant positive correlation with emotional intelligence (*r* = 0.462, *p* < 0.01); combat sports was significantly correlated with self-esteem (*r* = 0.406, *p* < 0.01); emotional intelligence was significantly correlated with undergraduate students’ subjective well-being (*r* = 0.504, *p* < 0.01); self-esteem was significantly correlated with undergraduate students’ subjective well-being (*r* = 0.453, *p* < 0.01); and emotional intelligence was significantly correlated with self-esteem (*r* = 0.394, *p* < 0.01). In summary, significant correlations exist among all variables, providing preliminary evidence for the hypotheses proposed in this study.

**Table 3:** Descriptive statistics and correlations for primary variables.

Variable	M	S.D.	Skew	Kurt	MBE	PSS	PR	QOL
CS	3.504	0.869	−0.381	−0.466	1			
SWB	3.162	0.743	−0.17	−0.606	0.534**	1		
EI	3.516	0.668	−0.667	0.311	0.462**	0.504**	1	
SE	2.611	0.700	−0.2	−1.052	0.406**	0.453**	0.394**	1

*n* = 1,304; M, mean; S.D., standard deviation; Skew, Skewness; Kurt, Kurtosis. *p < 0.05 **p < 0.01; CS, combat sports; SWB, subjective well-being; EI, emotional intelligence; SE, self-esteem.

To better examine the impact of combat sports on undergraduate students’ subjective well-being, this study employed one-way analysis of variance (ANOVA) to investigate the differences in subjective well-being across varying exercise intensity levels. As demonstrated in the Table 4 below, all exercise intensity groups exhibited significant differences in subjective well-being (*p* < 0.05), indicating that different exercise intensity levels differentially affect subjective well-being. Exercise intensity demonstrated significant effects on subjective well-being at the 0.01 level (*F* = 228.688, *p* = 0.000), suggesting that participation in moderate to high-intensity physical and mental exercise contributes more effectively to enhancing undergraduate students’ subjective well-being.

**Table 4:** Anova results of the effects of combat sports on undergraduate students’ subjective well-being.

Variable	Exercise amount (M + S.D.)			*F*	*p*
	Mild exercise (*n* = 472)	Moderate exercise (*n* = 387)	Intense exercise (*n* = 445)		
Subjective well-being	51.454 ± 12.946	59.556 ± 10.556	68.612 ± 12.602	228.688	0.000**

*p < 0.05 **p < 0.01.

### 5.4 Analysis of mediation effects

To verify the chain mediation effects of emotional intelligence and self-esteem in the relationship between combat sports and undergraduate students’ subjective well-being, AMOS 24.0 software was employed to conduct goodness-of-fit analysis for the conceptual chain mediation model. Following the mediation effect testing procedures proposed by Marsh et al. (2014), Table 5 presents the standard results of fit indices: *χ*^2^/df < 3, RMSEA < 0.08, CFI > 0.9, GFI > 0.9, NFI > 0.9, TLI > 0.9. The model parameters met the fit requirements, indicating that the mediation model conceptualization for combat sports and undergraduate students’ subjective well-being is reasonable.

**Table 5:** Questionnaire model fitting indicators.

	*X* ^2^	df	*χ*^2^/df	RMSEA	CFI	GFI	NFI	TLI	IFI
Model	33.325	164	2.020	0.028	0.986	0.975	0.973	0.984	0.986

RMSEA, root mean square error of approximation; CFI, comparative fit index; GFI, goodness-of-fit index; NFI, normed fit index; TLI, Tucker-Lewis index. IFI, increased fit index.

**Figure 2:**
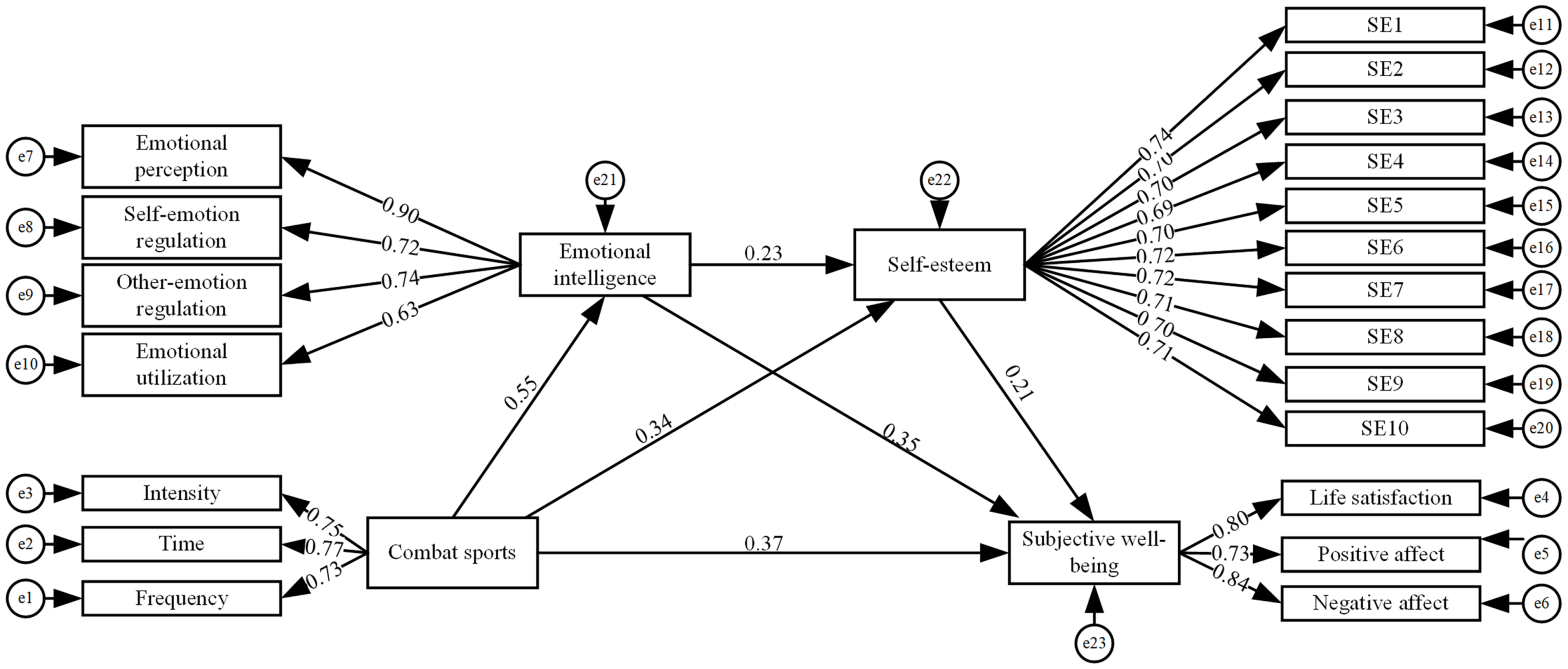
Intermediary model diagram.

As illustrated in Figure 2, the standardized path coefficient for combat sports → undergraduate students’ subjective well-being was significant (*β* = 0.371, *p* < 0.001), demonstrating that combat sports exert a significant positive influence on undergraduate students’ subjective well-being, thus supporting hypothesis H1. The path coefficients for combat sports → emotional intelligence (*β* = 0.553, *p* < 0.001) → undergraduate students’ subjective well-being (*β* = 0.34, *p* < 0.001) were significant, indicating that emotional intelligence mediates the relationship between combat sports and undergraduate students’ subjective well-being, thus supporting hypothesis H2. The path coefficients for combat sports → self-esteem (*β* = 0.351, *p* < 0.001) → undergraduate students’ subjective well-being (*β* = 0.206, *p* < 0.001) were significant, demonstrating that self-esteem mediates the relationship between combat sports and undergraduate students’ subjective well-being, thus supporting hypothesis H3. The path coefficients for combat sports → emotional intelligence (*β* = 0.553, *p* < 0.001) → self-esteem (*β* = 0.23, *p* < 0.001) → undergraduate students’ subjective well-being (*β* = 0.206, *p* < 0.001) were significant, indicating that emotional intelligence and self-esteem exhibit chain mediation effects between combat sports and undergraduate students’ subjective well-being, thus supporting hypothesis H4.

Bias-corrected nonparametric percentile Bootstrap methods were employed to assess the significance of individual mediation effects and confirm the mediating roles of emotional intelligence and self-esteem. Hayes recommended that Bootstrap mediation effect testing should include at least 1,000 resampled datasets from the original sample (Hayes, 2009). Bootstrap mediation effect testing results indicate that indirect effects are established when Bootstrap confidence intervals (CI) do not contain zero (Zhao et al., 2010). This study conducted mediation effect testing using 5,000 Bootstrap samples to determine 95% confidence intervals (CI).

As shown in Table 6, the direct effect of combat sports on undergraduate students’ subjective well-being was significant [direct effect = 0.461, 95% CI (0.343, 0.593)]. Indirect effects included three significant mediation pathways: combat sports → emotional intelligence → subjective well-being [indirect effect = 0.234, 95% CI (0.172, 0.311)]; combat sports → self-esteem → subjective well-being [indirect effect = 0.090, 95% CI (0.057, 0.132)]; and combat sports → emotional intelligence → self-esteem → subjective well-being [indirect effect = 0.033, 95% CI (0.017, 0.054)]. In summary, the Bootstrap 95% confidence intervals for all three mediation pathways excluded zero, indicating that the various path coefficients in this model were significant. These findings demonstrate that emotional intelligence and self-esteem mediate the relationship between combat sports and undergraduate students’ subjective well-being, with emotional intelligence and self-esteem exhibiting chain mediation effects.

**Table 6:** Test results of mediation effects.

Effect	Parameter	Estimate	BootSE	Bootstrap LLCI	Bootstrap ULCI
Direct effect	CS → SWB	0.461	0.064	0.343	0.593
Indirect effect	CS → EI → SWB	0.234	0.035	0.172	0.311
	CS → SE → SWB	0.090	0.019	0.057	0.132
	CS → EI → SE → SWB	0.033	0.009	0.017	0.054
Total effect	CS → EI → SE → SWB	0.818	0.054	0.712	0.925

The Bootstrap sample size is set at 5000.

## 6 Discussion

This study examined the impact of combat sports on undergraduate students’ subjective well-being, with particular emphasis on analyzing the chain mediation effects of emotional intelligence and self-esteem. The results demonstrated that combat sports exhibit a significant relationship with undergraduate students’ subjective well-being, with Wushu Sanda, Taekwondo, and Kickboxing all significantly and positively predicting undergraduate students’ subjective well-being, consistent with previous research findings (Zhou, 2025; Tadesse, 2017). Although Wushu Sanda, Taekwondo, and Kickboxing differ in their technical systems, they all possess high-intensity, multidimensional exercise characteristics. Sanda’s integrated “kicking, punching, and throwing” technical system requires comprehensive full-body coordination, Taekwondo’s precise leg techniques and footwork transitions emphasize the unity of lower extremity strength and flexibility, while Kickboxing’s combination techniques using fists, legs, knees, and elbows pursue fluidity in offensive-defensive transitions. These complex technical movements during high-intensity training can more effectively promote substantial neurotransmitter release. Particularly during high-intensity confrontational training, combat sports require participants to complete complex technical decisions and physical execution within extremely short timeframes. This highly integrated movement significantly promotes brain secretion of neurotransmitters such as endorphins and dopamine, with the release of these happiness hormones directly enhancing individuals’ positive emotional experiences (Harwood-Gross et al., 2020; Babiloni et al., 2010). Simultaneously, regular combat sports participation helps regulate cortisol levels (de Oliveira et al., 2022; Slimani et al., 2018), reducing stress responses and creating favorable physiological foundations for subjective well-being enhancement. Compared to traditional aerobic exercise, combat sports emphasize the cultivation of technical, strategic, and immediate response capabilities. This complex cognitive engagement process enhances individuals’ self-perception abilities (Russo and Ottoboni, 2019). When undergraduate students gradually master technical movements and improve competitive performance through training, they experience pronounced achievement and competence feelings. This positive self-evaluation directly promotes subjective well-being enhancement. The research further revealed that participation in high-intensity combat sports more effectively enhances undergraduate students’ subjective well-being. This enhancement manifests not only in improved physical function but also across various aspects of undergraduate students’ daily lives, promoting the development of long-term exercise habits, thereby continuously maintaining physical health status, enhancing undergraduate students’ self-confidence, promoting emotional stability, and consequently enhancing overall life satisfaction and well-being.

Combat sports not only directly influence undergraduate students’ subjective well-being but also indirectly affect subjective well-being through positive impacts on emotional intelligence, consistent with previous research findings (Oh et al., 2025). Enhancement of emotional intelligence capabilities develops significantly within the specialized context of combat sports. Existing research demonstrates that individuals exhibit more pronounced psychological characteristics and significantly elevated emotional intelligence levels following combat sports participation (Szabo and Urbán, 2014). In high-intensity, high-pressure confrontational exercise environments, participants must rapidly and accurately identify and regulate complex emotional states. This repetitive emotional practice effectively promotes the development of capabilities across all emotional intelligence dimensions (Kopp and Jekauc, 2025). Combat sports create increasingly variable and challenging emotional contexts where participants must cope with multiple intense emotions including fear, excitement, frustration, and anger during training and competition processes. Participants must adjust to optimal competitive states based on real-time circumstances while continuously maintaining emotional stability. Victory in combat sports is not determined by any single athletic capability but represents a concentrated manifestation of comprehensive qualities including physical fitness, technical proficiency, tactical knowledge, psychological qualities, and intellectual capacity. Therefore, combat sports are more conducive to cultivating undergraduate students’ emotional perception, self-emotional regulation, others’ emotional regulation, and emotional utilization capabilities, consistent with previous research findings (Zvi and Lavi, 2025). Emotional intelligence demonstrates a significant positive correlation with subjective well-being, consistent with previous research (Caballero-García and Ruiz, 2025). Individuals with high emotional intelligence can more accurately identify and understand emotional states in themselves and others. This emotional awareness assists individuals in conducting more objective and positive evaluations of life events. Furthermore, enhanced emotional regulation capabilities enable individuals to more effectively manage negative emotions while maintaining positive affective states. Research reveals that emotional intelligence influences subjective well-being by avoiding emotional exhaustion, increasing positive emotional experiences, and reducing negative emotional experiences (Extremera and Rey, 2016). This emotional regulation mechanism represents a critical pathway for subjective well-being enhancement.

Simultaneously, combat sports can indirectly influence undergraduate students’ subjective well-being through positive effects on self-esteem. First, skill acquisition in combat sports exhibits distinct stage-based and visualizable characteristics. The progression from basic movements to complex techniques provides individuals with clear feedback regarding capability enhancement, and this accumulation of competence constitutes an important foundation for self-esteem development. Second, compared to other sports, combat sports place greater emphasis on individual courage, perseverance, and self-control capabilities. During processes of facing opponents, enduring physical contact, and tolerating training intensity, participants must continuously push beyond psychological comfort zones. These experiences of self-challenge and transcendence demonstrate significant effects on self-esteem enhancement, consistent with previous research findings (Bojanić et al., 2019). Finally, Rosenberg’s self-esteem theory posits that self-esteem, as a core component of individual self-concept, directly influences individuals’ overall life evaluations and emotional experiences (Monteiro et al., 2022). Combat sports provide multiple positive experiences including skill mastery, physical fitness improvement, and challenge resolution, offering rich material for individuals to construct positive self-cognitions. When undergraduate students gradually master technical movements, enhance physical capabilities, and overcome psychological fears through combat sports participation, these successful experiences become internalized as positive cognitions regarding personal abilities and worth, thereby elevating self-esteem levels, consistent with previous research findings (Kostorz et al., 2017). Furthermore, individuals with high self-esteem tend to engage in more positive attributions and interpretations of life events. They more readily attribute positive events to internal and stable factors while attributing negative events to external and temporary factors. This attribution pattern facilitates maintenance of positive life evaluations. Simultaneously, high self-esteem individuals hold more optimistic expectations regarding the future, believing in their capability to address various challenges and difficulties. This optimistic attitude directly enhances life satisfaction. At the emotional level, self-esteem influences individuals’ emotional regulation capabilities and emotional experience patterns. Individuals with higher self-esteem demonstrate greater proficiency in maintaining positive emotional states, recover more rapidly from negative emotions, and experience positive emotions such as pride, satisfaction, and happiness more readily (Brown and Marshall, 2001). This positive bias in emotional experience constitutes an important component of subjective well-being.

A relatively stable systematic association exists between undergraduate students’ emotional intelligence and self-esteem, consistent with previous research findings (Miezah et al., 2025). The confrontational and competitive nature of combat sports requires participants to continuously identify opponents’ emotional states and attack intentions during training while simultaneously regulating their own negative emotions such as tension and anxiety. This process directly promotes individual emotional intelligence enhancement. When undergraduate students possess higher emotional intelligence, they can approach challenges in life and learning with more positive and stable mindsets, thereby improving subjective well-being levels, consistent with previous research findings (Sánchez-Álvarez et al., 2016). As an important cognitive resource, emotional intelligence enables undergraduate students to approach challenges in combat sports from positive perspectives, viewing failures as growth opportunities, reducing self-negation, and appropriately expressing emotions during social interactions to receive positive feedback from peers and coaches. This process enhances affirmation and identification of personal value, strengthening self-worth perceptions and elevating self-esteem levels. When individuals possess greater emotional intelligence, they experience more respect, support, and understanding emotionally, achieve higher internal satisfaction levels, and develop stronger capabilities to resist adversity, resulting in higher self-esteem levels, consistent with previous research findings (Bibi et al., 2016). Higher self-esteem subsequently makes undergraduate students more inclined to view life optimistically, more willing to actively participate in various activities, and more proactive in establishing positive relationships with others, consequently experiencing more positive emotions, improving life satisfaction, and ultimately promoting subjective well-being enhancement, consistent with previous research findings (Du et al., 2017). The results of this study further demonstrate that combat sports can collectively influence undergraduate students’ subjective well-being through chain mediation effects of emotional intelligence and self-esteem.

## 7 Research implications and limitations

### 7.1 Research implications

Existing research on combat sports has predominantly focused on practical intervention effectiveness assessment, while systematic theoretical exploration of its underlying mechanisms remains relatively scarce, particularly in-depth analyses based on SDT are notably insufficient. This study employs SDT as a theoretical framework to systematically examine the mechanisms through which combat sports influence undergraduate students’ subjective well-being via the chain mediation effects of emotional intelligence and self-esteem, providing meaningful theoretical extensions for SDT applications in the field of sport psychology. The core tenet of SDT posits that the satisfaction of three basic psychological needs, namely autonomy, competence, and relatedness, constitutes the key factor driving intrinsic motivation and promoting subjective well-being. The research findings indicate that combat sports can satisfy these basic psychological needs through multiple pathways. First, during the training process, students experience significant enhancement of competence through skill advancement and self-breakthrough. Second, social interactions and self-expression within group training environments effectively fulfill relatedness needs. Meanwhile, the marked improvement in emotional intelligence enables students to more precisely identify, understand, and regulate their emotional states, enhancing self-awareness and emotional control capabilities, thereby promoting the full development of autonomy. This enhancement of emotional management capabilities itself becomes an important source of self-esteem strengthening. This complete chain of effects powerfully validates and deepens the core theoretical hypothesis of SDT regarding how basic psychological need support promotes subjective well-being. More importantly, the chain mediation pathway of emotional intelligence and self-esteem revealed in this study exhibits clear temporal characteristics, with emotional intelligence as a procedural psychological resource developing first, subsequently promoting the formation and consolidation of self-esteem as an outcome psychological resource. This finding elucidates the bridging mechanism of emotional regulation capabilities and self-esteem in the process by which combat sports promote subjective well-being. It not only provides empirical support and mechanism refinement for SDT’s theoretical framework regarding how psychological need satisfaction promotes well-being, but also offers theoretical guidance for future research in designing combat sports-based psychological intervention programs.

At the practical level, this study provides novel approaches for undergraduate students’ mental health promotion initiatives. First, traditional mental health interventions predominantly employ passive approaches such as counseling and therapy, while this study confirms the effectiveness of combat sports as an active intervention strategy. When enhancing undergraduate students’ subjective well-being levels, comprehensive intervention measures should be adopted, considering the integrated effects of combat sports, emotional intelligence, and self-esteem factors to create positive living environments and healthy lifestyles for undergraduate students. Second, from an educational practice perspective, understanding the chain mediation effects of emotional intelligence and self-esteem facilitates optimization of combat sports teaching and training methodologies. Educators can consciously integrate emotional skills training into training processes, including emotional identification exercises, stress management techniques, and frustration coping strategies, thereby maximizing combat sports’ promotional effects on subjective well-being. Finally, attention should be directed toward undergraduate students’ self-esteem development by creating positive and inclusive sports atmospheres, avoiding harm to students’ self-esteem through excessive competition or inappropriate evaluations, enabling students to gradually establish self-confidence through combat sports participation. Regular comprehensive health education and psychological counseling can be implemented to promote more holistic psychological and social development, thereby increasing positive emotions and encouraging stronger self-confidence in daily life to enhance subjective well-being.

### 7.2 Research limitations

Despite providing compelling evidence for the positive effects of combat sports, emotional intelligence, and self-esteem on enhancing undergraduate students’ subjective well-being, this study has several important limitations that warrant explicit acknowledgment. First, the limitations of cross-sectional research design. This study employed a cross-sectional research design to explore the mechanisms through which combat sports influence undergraduate students’ subjective well-being. While this approach effectively examines associations between variables and mediation pathways, it inherently constrains the ability to make causal inferences and determine directionality, failing to capture the temporal developmental processes of psychological benefits, including their cumulative, persistent, and threshold characteristics. Future research should adopt longitudinal tracking designs to observe the developmental trajectories of psychological variables before and after combat sports participation through multi-timepoint measurements, clarifying temporal sequences to provide stronger evidential support for causal inference. Alternatively, randomized controlled trial designs could be implemented to randomly assign groups while controlling confounding variables, establishing rigorous causal relationships and exploring differential effects of various training parameters on psychological benefits. Second, limitations in dose–response relationship analysis. Although this study preliminarily explored the relationship between different exercise volumes and subjective well-being through the PARS-3 scale and univariate analysis of variance, confirming that moderate to high-intensity combat sports are more conducive to subjective well-being enhancement, this analysis remains insufficient in depth. The PARS-3 provides a composite physical activity score based on frequency, intensity, and duration, but cannot distinguish the independent contributions of these different dimensions to psychological benefits. The study lacks refined analysis of specific training parameters, such as the differential effects of various training durations, training frequencies, training intensity types, and session durations. Future research should employ multidimensional dose measurement approaches to systematically compare the psychological effects of different training parameter combinations, providing precise guidance for determining the optimal parameter configuration for psychological benefits of combat sports. Third, limitations in research scope. This study involved only three types of combat sports, namely Wushu sanda, taekwondo, and kickboxing, without including other types such as judo, boxing, and mixed martial arts. Different combat sports exhibit significant variations in technical characteristics, training intensity, and cultural backgrounds, potentially generating distinct psychological effects, warranting refined research designs in future studies. Simultaneously, the research sample was limited to universities in Sichuan Province, China, which cannot adequately represent undergraduate student populations from different geographical regions, cultural backgrounds, and economic development levels, thereby constraining the generalizability of the research findings. Future research could enhance the comprehensiveness of conclusions by expanding the geographical scope of sample collection.

## 8 Conclusion

This study revealed the impact of combat sports on undergraduate students’ subjective well-being and conducted in-depth analysis through the chain mediation effects of emotional intelligence and self-esteem. Results indicated that combat sports exert positive influences on undergraduate students’ subjective well-being. Undergraduate students who regularly participate in combat sports not only demonstrate higher subjective well-being but also achieve significant improvements in emotional intelligence and self-esteem. The research found that emotional intelligence plays a significant mediating role between combat sports and subjective well-being. Participation in combat sports significantly enhances undergraduate students’ emotional intelligence levels. The promoting effects of combat sports on emotional intelligence manifest across multiple dimensions including emotional perception, understanding, regulation, and utilization, providing rich practical opportunities for emotional management. Self-esteem similarly plays a significant mediating role between combat sports and subjective well-being. Mastery of combat sports skills provides individuals with sources of capability confidence, enhancing self-efficacy. Successful experiences and peer recognition during sports participation elevate individuals’ social value perceptions, subsequently promoting self-esteem enhancement and ultimately fostering subjective well-being improvement. This study represents the first verification of the chain mediation effects of emotional intelligence and self-esteem between combat sports and undergraduate students’ subjective well-being. Combat sports not only improve undergraduate students’ subjective well-being but also achieve comprehensive subjective well-being enhancement through effective utilization of emotional intelligence and strengthened self-esteem levels, maintaining positive life attitudes. The chain mediation model proposed in this study integrates the relationships among emotional intelligence, self-esteem, and undergraduate students’ subjective well-being, providing new perspectives for more comprehensive understanding of how combat sports influence undergraduate students’ subjective well-being through internal mechanisms. This finding not only enriches existing literature but also provides theoretical foundations for combat sports applications in undergraduate student health promotion domains.

## Data Availability

The datasets presented in this article are not readily available because the dataset in this study has the following limitations: the sample is drawn exclusively from college students in a specific region with a relatively concentrated age range (18–22 years), which limits the generalizability of findings to other age groups and cultural backgrounds; the cross-sectional survey design prevents the establishment of causal relationships between variables and can only reveal associations; all measurements rely on participants’ self-reports, which may introduce social desirability bias, recall bias, and other subjective evaluation biases. Requests to access the datasets should be directed to QY, yqq177123456@163.com.
